# Eosinophilic Cellulitis (Wells Syndrome): A Clinicopathologic Diagnosis in an Elderly Patient

**DOI:** 10.7759/cureus.113739

**Published:** 2026-07-31

**Authors:** Anja C Fernandez Placencia, Sigrid G Fernandez, Alex Ventura Leon, Paul G Gomez, Letty Probasco

**Affiliations:** 1 Internal Medicine, Universidad Ricardo Palma, Lima, PER; 2 Medicine, University of Maryland, College Park, USA; 3 Pathology, Cayetano Heredia Hospital, Lima, PER; 4 Milken Institute School of Public Health, The George Washington University, Washington, D.C., USA; 5 Rheumatology, Lehigh Valley Health Network, Allentown, USA

**Keywords:** eosinophilic cellulitis, peripheral eosinophilia, pseudocellulitis, skin biopsy, wells disease

## Abstract

Eosinophilic cellulitis, also known as Wells syndrome, is a rare inflammatory dermatosis that may clinically mimic bacterial cellulitis and contribute to diagnostic delay. We report the case of a 72-year-old woman with a history of systemic lupus erythematosus and other autoimmune comorbidities who initially presented with bilateral lower extremity erythema and received antibiotic therapy for presumed cellulitis, with transient improvement. Three days after completing treatment, she developed recurrent pruritic erythematous plaques involving both upper and lower extremities, accompanied by edema and pain, without significant improvement following antihistamine therapy. Laboratory studies revealed peripheral eosinophilia (absolute eosinophil count 1.07 × 10³/µL) and elevated erythrocyte sedimentation rate. Punch biopsy showed eosinophil-rich interstitial and perivascular inflammatory infiltrates consistent with eosinophilic cellulitis, and the patient subsequently improved with systemic corticosteroid therapy. This case highlights the importance of considering Wells syndrome in patients with recurrent cellulitis-like eruptions, particularly when response to antibiotic therapy is incomplete or transient. Early biopsy and clinicopathologic correlation remain essential for accurate diagnosis and timely management.

## Introduction

Wells syndrome, also known as eosinophilic cellulitis, was first described by Wells in 1971 as recurrent granulomatous dermatitis with eosinophilia. It is an uncommon inflammatory dermatosis of uncertain pathogenesis characterized by heterogeneous clinical manifestations, suggestive but nonspecific histopathologic findings, a recurrent course, and variable response to therapy [1]. Eosinophilic cellulitis predominantly affects adults and demonstrates no clear ethnic or gender predilection. Clinically, it typically presents acutely over two to three days as solitary or multiple infiltrated erythematous plaques resembling cellulitis or urticaria [2].

The exact etiology of Wells syndrome remains unclear. Although many reported cases are idiopathic, others have been associated with triggering events or chronic underlying disorders, supporting the hypothesis that Wells syndrome may represent a reactive eosinophilic inflammatory process rather than a distinct disease entity [3]. Proposed triggers include arthropod bites, infections, medications, vaccinations, biologic therapies, malignancies, and autoimmune diseases [4]. Current evidence suggests that eosinophilic activation and recruitment into the skin are mediated through a predominantly T helper 2 (Th2)-driven immune response, with interleukin-5 playing a central role in eosinophil proliferation, activation, and tissue migration [3]. The reported efficacy of anti-interleukin-5 therapy in refractory cases further supports the role of eosinophilic cytokine dysregulation in disease pathogenesis [4].

Lesions preferentially involve the extremities and are commonly associated with pruritus or burning sensations [5,6]. Peripheral blood eosinophilia is variably present and, although supportive, is not required for diagnosis of Wells syndrome. Diagnosis relies on clinicopathologic correlation, including recurrent cellulitis-like cutaneous lesions, exclusion of infectious and systemic mimics, and histopathologic demonstration of eosinophilic dermal infiltrates with or without flame figures [7]. Because clinical presentation frequently overlaps with infectious and inflammatory dermatoses, delayed or incorrect diagnosis may occur.

This diagnostic challenge may be even greater in patients with autoimmune or chronic inflammatory conditions, in whom Wells syndrome can mimic infectious cellulitis, vasculitis, or other inflammatory dermatoses encountered in rheumatologic practice [4]. We report the case of a 72-year-old woman with systemic autoimmune disease who presented with recurrent cellulitis-like eruptions and peripheral eosinophilia, ultimately diagnosed with Wells syndrome following histopathologic evaluation.

## Case presentation

A 72-year-old woman with a past medical history significant for systemic lupus erythematosus (SLE), perniosis, Raynaud phenomenon, psoriasis, psoriatic arthritis, hypertension, and deep vein thrombosis status post Greenfield filter placement presented to the emergency department with sudden-onset bilateral lower extremity erythema and swelling. The initial clinical impression was bilateral bacterial cellulitis, and empiric intravenous vancomycin therapy was initiated. She was subsequently discharged on oral clindamycin to complete a 10-day antibiotic course, with partial improvement, although residual erythema persisted at the time of discharge.

Three days after completing antibiotic therapy, the patient developed recurrent erythematous, edematous plaques associated with intense pruritus and pain involving the bilateral thighs and upper extremities. Symptoms were unresponsive to oral diphenhydramine. She denied recent insect bites, new medications, recent travel, or new detergents or topical products and had no personal history of atopy or known drug allergies.

Initial laboratory evaluation demonstrated peripheral eosinophilia, with an absolute eosinophil count of 1.07 × 10³/µL, and a mildly elevated erythrocyte sedimentation rate (ESR). Hemoglobin, total leukocyte count, platelet count, and C-reactive protein (CRP) of 3 mg/L were within normal limits. Complete blood count and inflammatory marker results are summarized in Table 1.

**Table 1:** Complete blood count, differential, and inflammatory markers.

Parameter	Result	Reference range
White blood cell count	6.7 × 10³/µL	4.0-11.0 × 10³/µL
Hemoglobin	14.2 g/dL	12.0-16.0 g/dL
Platelet count	210 × 10³/µL	150-450 × 10³/µL
Eosinophils	16%	0%-6%
Absolute eosinophil count	1.07 × 10³/µL	0.00-0.50 × 10³/µL
Erythrocyte sedimentation rate (ESR)	32 mm/hr	0-30 mm/hr
C-reactive protein (CRP)	3 mg/L	<10 mg/L

Rheumatologic testing revealed a positive antinuclear antibody (ANA) at a titer of 1:80 with a speckled pattern and a positive atypical perinuclear anti-neutrophil cytoplasmic antibodies (p-ANCA), whereas the extractable nuclear antigen (ENA) panel, myeloperoxidase (MPO), and proteinase-3 (PR3) antibodies were negative; complement levels were within normal limits. Rheumatologic laboratory findings are summarized in Table 2.

**Table 2:** Rheumatologic and autoimmune serologic workup. p-ANCA: perinuclear anti-neutrophil cytoplasmic antibodies

Parameter	Result	Reference range
Antinuclear antibody (ANA)	Positive, 1:80 speckled pattern	Negative (<1:80)
Extractable nuclear antigen (ENA) panel	Negative	Negative
Atypical p-ANCA	Positive	Negative
Myeloperoxidase (MPO) antibody	Negative	Negative
Proteinase-3 (PR3) antibody	Negative	Negative

Additional laboratory evaluation showed normal renal, hepatic, and thyroid function tests, and urinalysis was unremarkable. Prior diagnostic studies, including a ventilation-perfusion scan, electrocardiogram, chest radiograph, and bilateral lower extremity Doppler ultrasonography, were unrevealing.

Physical examination revealed erythematous infiltrated plaques involving the bilateral upper and lower extremities, with associated pitting edema and overlying dry, flaking skin (Figure 1). No palpable lymphadenopathy was identified, and the remainder of the physical examination was unremarkable.

**Figure 1:**
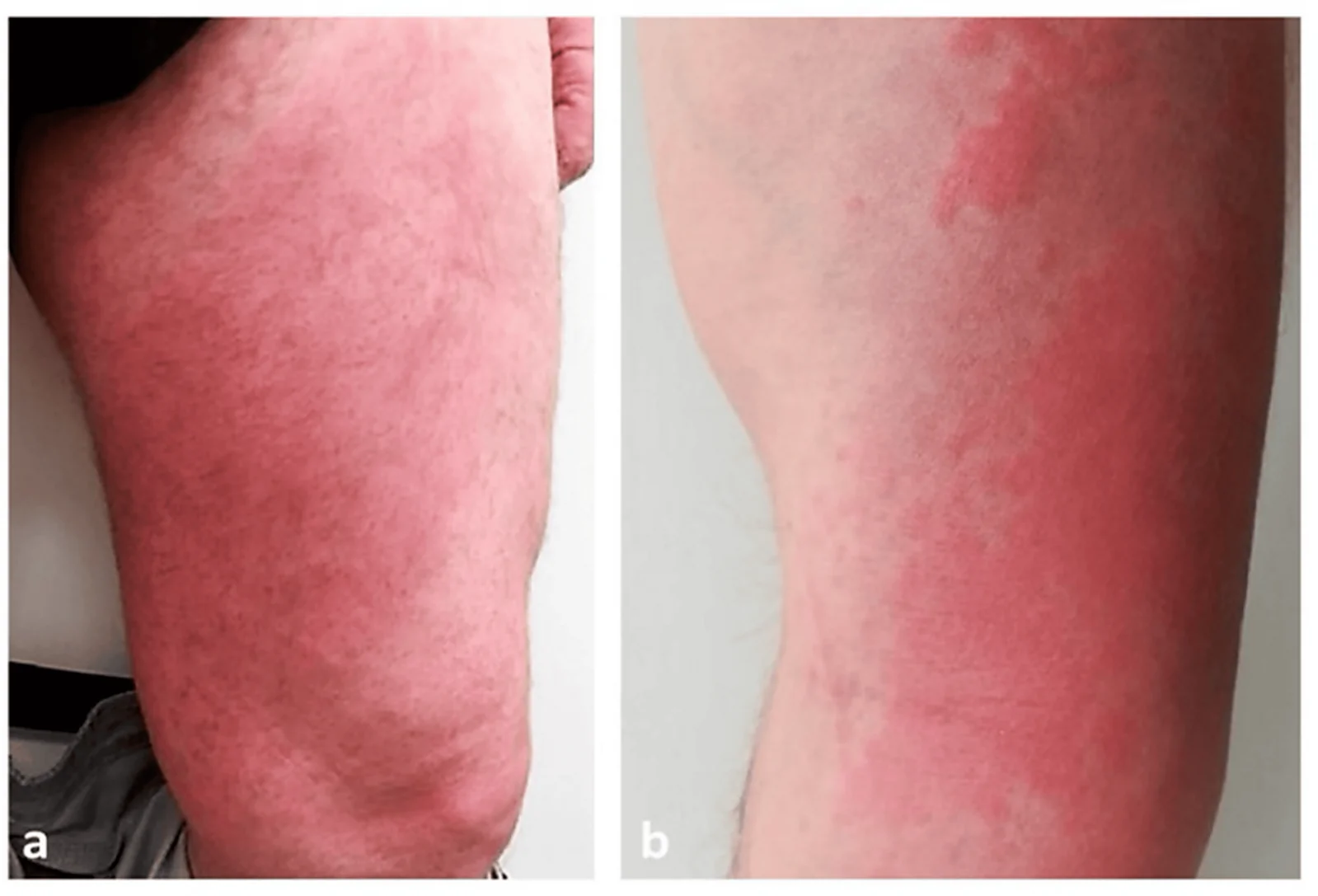
Clinical presentation of Wells syndrome. (a) Large erythematous infiltrated plaque involving the lateral thigh with poorly demarcated borders and associated edema, clinically resembling cellulitis. (b) Confluent erythematous plaque involving the medial thigh with irregular margins and surrounding edema, demonstrating the bilateral cellulitis-like eruption characteristic of Wells syndrome.

Given the recurrent cellulitis-like eruption, peripheral eosinophilia, and lack of sustained response to antibiotic therapy, a punch biopsy of an active lesion was performed. Histopathologic examination demonstrated superficial and mid-dermal interstitial and perivascular eosinophil-rich inflammatory infiltrates admixed with lymphocytes, accompanied by prominent dermal edema and eosinophilic degranulation. No evidence of leukocytoclastic vasculitis was identified. Although flame figures were not conspicuous, the overall histopathologic findings, in correlation with the clinical presentation, supported a diagnosis of Wells syndrome (Figure 2).

**Figure 2:**
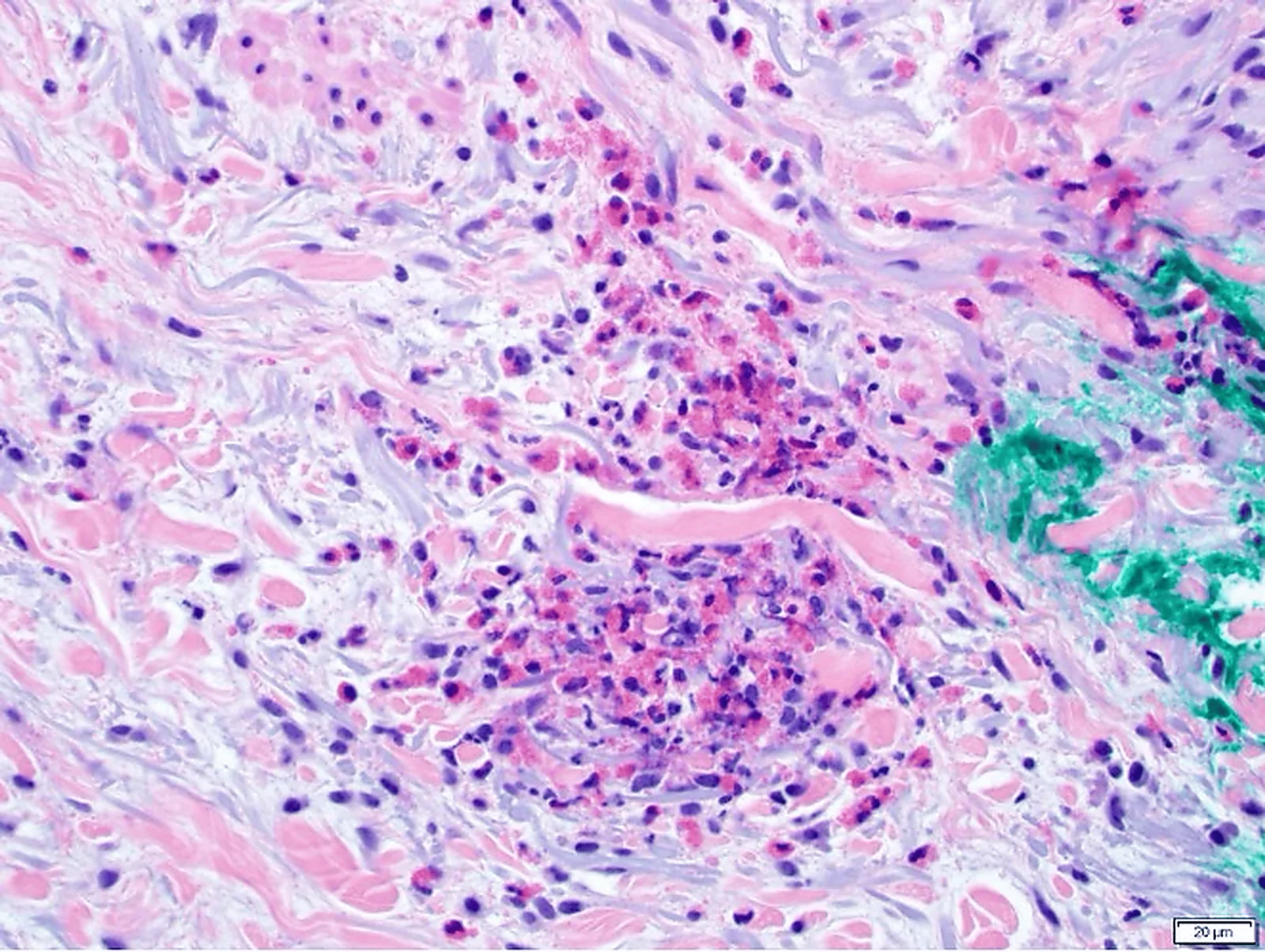
Histopathologic findings of Wells syndrome (hematoxylin and eosin stain, 400× magnification). Superficial and mid-dermal interstitial and perivascular eosinophil-rich inflammatory infiltrates accompanied by dermal edema and eosinophilic degranulation. No evidence of leukocytoclastic vasculitis was identified.

Integrating the clinical presentation with the laboratory and histopathologic findings, the differential diagnosis included recurrent bacterial cellulitis, eosinophilic granulomatosis with polyangiitis, urticarial vasculitis, drug eruption, and allergic contact dermatitis. These diagnoses were excluded based on the absence of compatible clinical features or exposure history, negative MPO and PR3 antibodies with normal complement levels, the absence of leukocytoclastic vasculitis on histopathology, and overall clinicopathologic correlation.

After establishing the diagnosis of Wells syndrome, treatment was initiated with prednisone 60 mg daily, tapered by 20 mg every five days, in combination with hydroxyzine 50 mg three times daily. This resulted in marked clinical improvement.

## Discussion

Wells syndrome, or eosinophilic cellulitis, is an uncommon inflammatory dermatosis characterized by recurrent cellulitis-like cutaneous eruptions accompanied by eosinophilic dermal infiltration on histopathology. The condition remains diagnostically challenging because its clinical manifestations frequently overlap with infectious cellulitis, urticarial disorders, vasculitis, drug eruptions, and other inflammatory dermatoses [1,7]. Our patient initially received broad-spectrum antibiotic therapy for presumed bilateral cellulitis with only transient improvement, followed shortly by recurrence of pruritic infiltrated plaques involving both upper and lower extremities. In the present case, recurrence of intensely pruritic plaques shortly after completion of antibiotic therapy, coupled with peripheral eosinophilia and eosinophil-rich dermal inflammation on biopsy, provided important clues supporting a diagnosis of Wells syndrome. This clinical course underscores the value of maintaining diagnostic suspicion when presumed cellulitis fails to demonstrate sustained improvement following appropriate antimicrobial treatment [1,4,6,7].

Although the exact pathogenesis of Wells syndrome remains incompletely understood, increasing evidence supports a predominantly eosinophil-mediated inflammatory process driven by Th2 cytokine signaling, particularly interleukin-5 [3,4]. Proposed triggers include infections, medications, arthropod bites, vaccinations, biologic therapies, malignancies, and autoimmune diseases [4]. In the present case, the coexistence of SLE, Raynaud phenomenon, perniosis, psoriasis, and psoriatic arthritis highlights the potential role of underlying immune dysregulation in promoting eosinophilic activation and cutaneous inflammation. Autoimmune-associated Wells syndrome has been described previously, although it remains relatively uncommon and may further complicate diagnostic evaluation because clinical findings can mimic vasculitic or connective tissue disease-related skin manifestations [1,4].

Peripheral eosinophilia, while not universally present, may provide an important diagnostic clue and has been reported in approximately 50% of cases [6,7]. Our patient demonstrated eosinophilia comprising 16% of circulating leukocytes with an absolute eosinophil count of 1.07 × 10³/µL. Histopathologic examination remains essential for diagnosis and typically demonstrates dermal eosinophilic infiltrates with variable edema and eosinophilic degranulation; flame figures may be observed but are neither pathognomonic nor universally present [5,6]. Absence of conspicuous flame figures should not exclude the diagnosis when compatible clinicopathologic features are present. In this case, punch biopsy demonstrated superficial and mid-dermal eosinophil-rich interstitial and perivascular inflammatory infiltrates with associated dermal edema and eosinophilic degranulation, without evidence of leukocytoclastic vasculitis. These findings, together with the recurrent cellulitis-like clinical presentation and peripheral eosinophilia, supported the diagnosis of eosinophilic cellulitis.

Systemic corticosteroids remain the mainstay of therapy and are generally associated with rapid clinical improvement [8]. Our patient demonstrated rapid clinical improvement following initiation of prednisone therapy with gradual tapering. However, recurrence is common, particularly during corticosteroid tapering, and refractory cases may require alternative therapies, including dapsone, cyclosporine, methotrexate, and antihistamines [8,9]. More recently, biologic agents targeting eosinophilic pathways, including anti-interleukin-5 therapies, have been reported in selected refractory cases [4].

This case highlights the diagnostic challenge posed by Wells syndrome in an elderly patient with multiple autoimmune comorbidities presenting with recurrent cellulitis-mimicking plaques. The combination of transient response to antibiotics, peripheral eosinophilia, and eosinophil-rich dermal inflammation ultimately established the diagnosis. Because recurrence is common and clinical findings frequently overlap with infectious cellulitis, early dermatologic evaluation and skin biopsy are critical to avoid unnecessary antimicrobial exposure and delays in appropriate immunomodulatory treatment. Although long-term follow-up was unavailable, the patient’s prompt response to systemic corticosteroids was consistent with previously reported therapeutic outcomes.

## Conclusions

Wells syndrome is a rare eosinophilic dermatosis that may closely mimic bacterial cellulitis and lead to diagnostic delay. This case highlights the importance of considering Wells syndrome in patients with recurrent or treatment-refractory cellulitis-like eruptions, particularly in the presence of peripheral eosinophilia. Early dermatologic evaluation, clinicopathologic correlation, and skin biopsy remain critical for establishing the diagnosis, avoiding unnecessary antibiotic exposure, and initiating appropriate therapy.
